# Adults with Autism: Changes in Understanding Since DSM-111

**DOI:** 10.1007/s10803-020-04847-z

**Published:** 2021-01-20

**Authors:** Patricia Howlin

**Affiliations:** Institute of Psychiatry, Psychology and Neuroscience, King’s College, London, UK

**Keywords:** Autism, Adulthood, Outcomes, Lifetime trajectories

## Abstract

Over the past four decades there have been significant advances in our understanding of autism, yet services for autistic adults continue to lag far behind those for children, and prospects for employment and independent living remain poor. Adult outcomes also vary widely and while cognitive and language abilities are important prognostic indicators, the influence of social, emotional, familial and many other factors remains uncertain. For this special issue marking the 40th anniversary of DSM-III, the present paper describes the changing perspectives of autism in adulthood that have occurred over this period, explores individual and wider environmental factors related to outcome, and suggests ways in which services need to be changed to improve the future for adults living with autism.

In the four decades since the publication of DSM-III (American Psychiatric Association [APA] 1980) there have been many changes in our understanding of autism. We now know much more about how reliably to diagnose the condition; underlying genetic and neuropsychological processes are far better understood, and the quality of intervention research- at least with respect to young children- has greatly improved. Nevertheless, very many autistic adults, and their families, continue to face significant difficulties in their daily lives. To mark the 40th anniversary of DSM-III, the following article focuses principally on research conducted over the intervening period that has changed our understanding of autism in adulthood. Variables related to prognosis, challenges experienced in adulthood, and interventions that may help to reduce these are explored. The paper concludes with recommendations for improving quality of life for autistic adults and those living with or caring for them.

## Changes in Concepts of Autism in Adulthood Since the Publication of DSM-III

### Autism is Not a Childhood Disorder

When DSM-III appeared 40 years ago*, Infantile Autism* remained the formal diagnostic classification and autism was still considered as a predominantly childhood disorder. Even in the later revision (DSM-III-R, APA 1987), when the name was changed to Autistic disorder, most of the core symptoms focused on childhood characteristics, with little attention given to how the condition might manifest later in life. When DSM-III was published, despite a huge upsurge in research on autistic *children* at the time, only a handful of studies focused on adult life. One of the most significant of these was Kanner’s own follow-up (1973) of 11 adults whom he had first diagnosed in the 1940′s. A subsequent review (Lotter 1978) identified four other cohorts with “infantile autism” or “child psychosis” followed up into adolescence/young adulthood (Creak 1963; Eisenberg 1956; Lotter 1974; Rutter et al. 1967). In the decades following the publication of DSM-III there has been growing recognition that autism is a lifelong condition, extending throughout adolescence and adulthood into old age (Happé and Frith 2020). Nevertheless, even today, compared with the volume of research on autistic children, the relative number of adult studies remains very low. Thus, a 2017 review (Howlin and Magiati 2017) estimated that only around 3.5% of published research on autism involved adults, and the proportion of studies with a focus on adult supports and services is even lower (Bishop-Fitzpatrick et al. 2014; Shattuck et al. 2020).

### Autism is Not Inevitably Associated with Severe Cognitive and Language Impairments

At the time of publication of DSM-III, autism was generally viewed as being almost always associated with moderate to severe intellectual impairment and diagnostic criteria also required “gross deficits in language development”. Indeed, even by the time of the DSM-IV-Text revision (2000), it was noted that “In most cases there is an associated diagnosis of Mental Retardation”. However, recent evidence suggests that up to two thirds of individuals with autism have an IQ in the average range or above (Chiarotti and Venerosi 2020; MacKay et al. 2018). Similarly, around 60–75% of individuals do develop some useful speech (Gernsbacher et al. 2016; Rose et al. 2016; Tager-Flusberg and Kasari 2013; Wodka et al. 2013), and in the current version of DSM-5 (2013) there is no mention of delay in language development as a core diagnostic symptom.

The wide range of intellectual and language skills associated with autism was highlighted in Lorna Wing’s article (1981), “Asperger's syndrome: a clinical account”. This described a group of individuals, many of whom were adults, who, despite showing the core symptoms of autism were of at least average intellectual ability, often had good language, and tended to possess particularly well developed skills or interests in areas such as memory, mathematics, astronomy, history, geography, geology, etc. Wing argued that the inclusion of Asperger’s disorder as a subtype of autism was necessary to draw attention to the problems of individuals who, although highly intelligent, were significantly affected by their condition and required understanding and appropriate support in order to help them thrive. The next two decades saw a marked increase in research on “higher ability” autistic individuals, and specific criteria for Asperger’s disorder appeared in ICD-10 (WHO 1992) and DSM-IV (APA 1994). Although the diagnostic category of “Asperger’s Disorder” did not survive into DSM-5, its introduction at the time was important in describing the heterogeneity of cognitive and linguistic abilities in autism and highlighting the significant potential of many autistic adults.

### Adulthood is Associated with Improvements as well as Difficulties

Although references to autism as a “devastating condition” still appear from time to time (e.g. EU Healthcare and Social Care News, Feb 2020), it is evident that very many individuals are able to live fulfilled and productive lives. Kanner (1973) was one of the first clinicians to describe a group of autistic adults who, despite the severity of their symptoms when younger, were subsequently functioning remarkably well, and *mingling, working and maintaining themselves in society*. In particular he noted that higher achievers were characterized by greater self-awareness of their difficulties and the ability to use their special skills and interests in ways that enhanced social inclusion and led to success in education and employment. Asperger (1944; translated by Frith 1991, p. 88) went so far as to suggest that *able autistic individuals can rise to eminent positions and perform with such outstanding success that one may even conclude that only such people are capable of certain achievements*. Subsequent studies have also described a wide range of autistic talents, with reviews suggesting that around two-thirds of individuals possess special strengths or skills (Meilleur et al. 2015).

While outcomes are highly variable, most autistic individuals show steady improvements as they make the transition from childhood to young adulthood. Severity of autism symptoms tends to reduce; social skills often improve, particularly around adolescence; many who are initially non-verbal acquire some level of useful speech, and ritualistic behaviors and sensory sensitivities typically become less impairing with age (Le Couteur and Szatmari 2015). Adaptive functioning and independence skills also tend to show improvements over time (e.g. Gillespie-Lynch et al. 2012), and several longitudinal studies report stability or modest increases in IQ (Anderson et al. 2014; Howlin et al. 2014; Kim et al. 2018; Lord et al. 2020; Pickles et al. 2020; Simonoff et al. 2019). It is also important to bear in mind that most long-term follow-up data derive from cohorts who were diagnosed with autism as young children, 20 or more years ago. In past decades, the heterogeneity of autism was not fully recognized and hence individuals of higher ability may have been less likely to be recruited into these early follow-up studies. This potential bias means that we still know relatively little about the achievements of individuals in whom the presentation of early symptoms is more subtle; nor do we know how their life experiences may have helped to enhance or limit success as adults.

## Social Outcomes and Quality of Life for Adults with Autism

Despite many studies demonstrating positive changes in *individual* functioning with age, it is evident that very many individuals with autism, regardless of IQ and ability, remain highly disadvantaged as adults (Howlin and Magiati 2017). A recent meta-analysis (Mason et al. 2020) identified 17 studies (from 8074 records searched) involving 1076 participants. Pooled estimates of outcomes indicated that approximately18% of participants were rated as having a “Good” outcome in terms of employment, social relationships and independent living; 28% were rated as having a “Fair” outcome”(i.e. remaining relatively dependent, but in some form of supported employment and with some social activities outside the home); 51% were rated as “Poor”. A previous meta-analysis (Steinhausen et al. 2016; 15 studies, 828 individuals) produced similar estimates (20% “Good”; 31% “Fair”; 48% “Poor”). These figure suggest that only around a fifth of participants in these studies could be considered to be well integrated within society and around half were described as having a poor or very poor outcome. Of additional concern is the fact that a comparison between earlier follow-up studies (i.e. pre 2000) and those conducted post 2000 (Howlin and Moss 2012), showed little improvement in overall outcomes, despite the expansion in specialist educational and intervention programs for autistic children over the same period.

In a series of studies, Taylor et al. (2014, 2015), Chan et al. (2018) have also highlighted the lack and/or poor quality of provision for young people with autism once they leave school. Even if post-school educational and work opportunities are available, only a minority (< 25%) of individuals remain engaged with these activities over time (Taylor et al. 2014, 2015). Females, people from more disadvantaged backgrounds and those with intellectual disability and/or behavioral difficulties are least likely to remain in education or employment, whilst greater, and more sustained, independence is associated with better daily living skills and (albeit marginally) with levels of service support.

It is not only in comparison with the general population that adults with autism fare poorly. Using data from the US National Longitudinal Transition Study-2 (NLTS2; n > 11,000 adults) Orsmond et al. (2013) compared rates of participation in social activities among young adults with autism and those with intellectual, emotional, behavioral or learning disabilities. Individuals in the autism group were significantly more likely never to see or get called by friends, never be invited to activities, and to be socially isolated. Data from the same source (Roux 2015; Roux et al. 2013; Shattuck et al. 2012) also indicated that the social, educational and employment outcomes of young adults with autism were poorer than those for individuals with other child-onset developmental disorders (speech/language impairments, learning, and intellectual disabilities). Although these group differences tend to disappear when participants are well matched for childhood IQ (Lord et al. 2020) it remains clear that autistic individuals have very low rates of participation in post-secondary education and employment. Thus, Roux (2015) found that 42% of autistic participants received no transition plan when they left school; 54% had no vocational or life-skills support as adults, and 26% had *no* support as adults. The situation was even worse for autistic individuals from socially disadvantaged, minority or low-income groups.

Together all these factors-limited independence in adulthood, lack of educational and employment opportunities, exclusion from peer support networks and an absence of specialized services, especially for individuals of average or above IQ- are suggested as contributing to a reduced quality of life (QoL) (Ayres et al. 2018; Lawson et al. 2020; Mason et al. 2019; van Heijst and Geurts 2015). Factors associated with a lower quality of life in autism include the presence of mental health problems and/or more autistic symptoms. A more positive QoL is correlated with being employed, receiving support and being in a close relationship. Autistic women tend to report a higher social QoL and men a higher physical QoL (Mason et al. 2019), but investigations of the association between QoL and other factors, such as intellectual ability or overall social functioning, have produced inconsistent findings (Kim and Bottema-Beutel 2019; Lord et al. 2020). It is also important to note that not all studies have found differences in QoL between adults with autism and those in the general population, (Hong et al. 2016; Moss et al. 2017; Oakley et al. 2020) although this may be at least partly due to the limitations of standard QoL measures (c.f McConachie et al. 2018, 2020).

## Is There Recovery from Autism?

While many follow-up studies have focused on the difficulties associated with adulthood, the outstanding achievements of some autistic adults have led to increased interest in those individuals who are described as having “optimal outcomes”, “very positive outcomes”, or even as showing “recovery” from autism. That is, as they move into later childhood or adolescence, they no longer show any overt symptoms of autism or other social or mental health problems (e.g. Anderson et al. 2014; Cederlund et al. 2008; Fein et al. 2013; Gillberg et al. 2016; Helles et al. 2017; Helt et al. 2008; Orinstein et al. 2015a, b). In each of these studies, the proportion of individuals reported to have lost all their autistic symptoms is small (generally less than 10%; Whiteley et al. 2019) and subsequent, more detailed data analyses have often indicated some persisting subtle difficulties in the domains of social-communication, cognition, and emotional recognition. There may also be certain mental health difficulties, particularly related to phobias and ADHD (Orinstein et al. 2015a, b). To date, there is little information on these individuals beyond adolescence or early adulthood; hence how many individuals do indeed show full “recovery” remains uncertain. Similarly, it has not yet proved possible to identify any distinctive variables that characterize individuals with an “optimal” or “very positive” outcome (Bölte 2014). For example, although individuals with “optimal”/“very positive outcomes” are typically of at least average IQ, many other autistic people of this cognitive level continue to have persisting difficulties. There are no data to show that any particular type or intensity of early of intervention is sufficient to predict later outcome (Orinstein et al. 2014) and, to date, there is little evidence that any specific environmental factors are related to “optimal” prognosis.

## The Impact of Individual Characteristics on Outcome

Since the early accounts of Kanner (1973) and Asperger (see Frith 1991) it has been noted that the development of useful speech and having an IQ in the average range are amongst the most reliable predictors of a more positive outcome in adulthood, and the importance of these variables has been confirmed in subsequent longitudinal studies (e.g. Anderson et al. 2014; Billstedt et al. 2007; Howlin et al. 2014; Lord et al. 2015; Pickles et al. 2020; Simonoff et al. 2019; Visser et al. 2017). Autistic children with moderate to severe cognitive impairments (IQ < 50) are unlikely become independent adults; most continue to need substantial support, and some may experience a deterioration in cognitive skills and/or increases in problem behaviors or autism symptomatology with age (Lord et al. 2015). Nevertheless, although autistic children with an IQ of at least 70 have a higher chance of achieving educational and occupational success as adults, several longitudinal studies of individuals of this cognitive level report only modest correlations between early cognitive and language scores and levels of adult attainment (Gray et al. 2012; Howlin et al. 2014; Lord et al. 2015). Moreover, IQ is not necessarily a good indicator of functional daily living skills (Alvares et al. 2020) and many individuals who are described as “high functioning” continue to experience significant social, emotional and/ or behavioral challenges in adulthood. Many have limited independence, continue to require considerable support and often remain living with parents, with little social participation outside the family (Lord et al. 2020; Orsmond et al. 2013).

As it has become increasingly evident that IQ alone is not a sufficiently reliable prognostic indicator, recent research has highlighted the need to identify mediators between IQ and outcome. For example, Simonoff et al. (2019) found that attendance at mainstream school had a significant long-term effect on outcome, independent of IQ. The authors suggest that being of higher ability may extend the range of experiences available to an individual, leading to greater opportunities for social inclusion and improvements in autism symptoms. However, as discussed below, many other factors can also make the difference between those who thrive in adulthood and those who do not.

### Mental Health

All too frequently in clinical services we see individuals who, during their childhood, have shown a reduction in autistic symptoms and who have initially made good progress, both academically and socially, but who then begin to experience mental health problems in mid- to later adolescence. Among such individuals, poor mental health often has a more negative impact on functioning than autism symptoms per se. However, reported prevalence of mental health problems in autism varies widely (from 20 to 25% to over 75%; Moss et al. 2015) and establishing accurate estimates remains a challenge (Moss et al. 2015). In particular, there is a lack of validated psychiatric assessment measures for people with autism (Brugha et al. 2015), especially those who are non-verbal and/or of low cognitive ability. Presentation of psychiatric symptoms may be atypical, and poor communication skills can limit the ability to describe abstract thoughts and emotions. Thus, diagnosis often depends on proxy accounts from parents or carers, which may not necessarily reflect the full extent of an individual’s difficulties (Sandercock et al. 2020). Nevertheless, it is now generally accepted that around 40% of autistic individuals experience a diagnosable mental health disorder at some stage in their lives (Hollocks et al. 2018). The most commonly reported problems are depression and anxiety related difficulties, including phobias, Post-Traumatic Stress Disorder (PTSD), and Obsessive–Compulsive Disorder (OCD); rates of ADHD are also high. In contrast, the occurrence of schizophrenia or severe psychosis appears to be relatively low (Hollocks et al. 2018; Lai et al. 2019; Lugo-Marin et al. 2019).

Studies exploring trajectories of mental health problems in adulthood are generally small and/or cross-sectional (Roestorf et al. 2019) and findings tend to vary according to participants’ age, gender, autism severity, intellectual level and the disorders studied (Lai et al. 2019; Uljarević et al. 2020). A recent longitudinal study (McCauley et al. 2020) of individuals followed from childhood into their mid-twenties, found that anxiety symptoms remained fairly stable, while ADHD symptoms decreased over time; depressive symptoms tended to increase from adolescence to early twenties, decreasing thereafter. Cross-sectional studies (e.g. Lever and Geurts 2016a, b; Uljarević et al. 2020) also suggest that severity and/or rates of mental health problems, particularly anxiety and depression, tend to be lower in middle-to-older age groups than in young adults.

Autism has been associated, in some studies, with an increased risk of suicide (Hirvikoski et al. 2016), suicidal ideation or suicide attempts (Cassidy et al. 2014, 2018). However, again, estimates vary across studies (Hedley and Uljarević 2018), and Croen et al. (2015) found that only 2% of the 1507 autistic individuals in their US database study were reported to have attempted suicide. Whatever the exact rates, it is clear that the risk of suicide, especially in individuals with autism who are cognitively and verbally able, is unacceptably high. Far more research is needed into the factors associated with suicide and for developing more reliable and effective ways of identifying and supporting those individuals who are at greater risk (Cassidy and Rodgers 2017).

Amongst other mental health problems, eating disorders appear to be elevated in autistic females, with some research suggesting that up to a third of women with eating disorders may have (often undiagnosed) autism (Mandy and Tchanturia 2015; Westwood and Tchanturia 2017). Autistic individuals with eating disorders also tend to have more severe symptoms and show a poorer response to treatment (Tchanturia et al. 2019).

There have been some reports of higher rates of substance or alcohol abuse in autism (Butwicka et al. 2017) although most existing research is based on very small or selective samples. Consequently, estimates of the numbers of autistic adults affected vary widely (< 1% to > 30%) and, at present, poor methodology and very heterogeneous samples make it impossible to establish accurate figures (Arnevik and Helverschou 2016; Ressel et al. 2020). Arnevik and Helverschou (2016) also highlight the often poor response to standard treatments for substance abuse among autistic individuals. Isenberg et al. (2019) stress the importance of early identification of problems; the need for treatments to take account of the communication and social difficulties, and poor motivation and insight, that characterize individuals with autism, as well as the need to find appropriate alternative social activities, and to engage parents or guardians in treatment.

The causes of mental health problems in autism remain poorly understood. Although genetic factors play a role (Tick et al. 2016), and rates of and emotional and behavioral problems are high from early childhood onwards (Stringer et al. 2020), the onset of new mental health difficulties in adult life often seems to be associated with major life events or transition points (such as leaving school, moving home, coping with college or starting employment (Hutton et al. 2008). It has been suggested that autistic individuals of higher intellectual ability may be more likely to develop mental health problems, especially related to depression and anxiety, than those of lower IQ. Although there is limited evidence to support this hypothesis (e.g. Rosen et al. 2018), Lord et al. (2019) found that young autistic adults (mean age 26 years) of average or above IQ, tended to show more depressive symptoms and negative emotions, and lower levels of well-being than those with IQ < 70. It may be that those individuals of higher IQ, who are more fully integrated in society, have greater awareness of their difficulties, as well as facing constant challenges in coping with the social, sensory and other demands of daily life. Accounts by higher ability autistic adults confirm that many feel under intense pressure to conform to the conventions of society and hence strive to “mask” or “camouflage” their differences. In turn, there is evidence that social camouflaging is strongly associated with mental health problems, particularly in autistic females (Beck et al. 2020), although the mechanisms underlying this relationship remain largely unknown (Lai et al. 2017; Mandy 2019).

Research into the impact of social factors on mental health problems has produced somewhat conflicting results. Thus, in a review by Smith and White (2020), disrupted social relationships and a perceived lack of social support were identified as being significantly associated with mental health problems in autistic adolescents and adults. Smith and White (2020) also concluded that individuals who wish for social contacts, but who remain lonely and/or socially isolated, are more likely to experience depression than those who express less desire for social contact. In contrast, an earlier study by Gotham et al. (2014) found that that adolescents and adults with the highest self-reported depressive symptoms tended to report both low social participation *and* low social motivation. In that study, the strongest link with depressive symptoms was the rate of self-perceived, autism-related impairments.

### Physical Health

It is now evident that many chronic health problems are significantly more common in autism than in the general population, and that these continue into old age (Bishop-Fitzpatrick and Kind 2017; Bishop-Fitzpatrick and Rubenstein 2019; Cashin et al. 2018; Croen et al. 2015; MacKay et al. 2018; Weir et al. 2020). Recent estimates of the total annual health care costs for individual with autism in the USA suggest that these are double the general population costs (Zerbo et al. 2019). Problems include immune conditions, gastrointestinal and sleep disorders, cardio-vascular disease, motor problems, seizures, obesity, hypertension, diabetes, stroke, Parkinson’s disease, and side effects from long-term medication use. Surveys of individuals with autism also show that they face many barriers in accessing health care. Among the most commonly cited difficulties are: lack of understanding of autism by healthcare staff; difficulties related to sensory sensitivity issues; “not knowing where (or how) to find help”, and negative experiences with professionals (Mason et al. 2019; Vogan et al. 2017).

High rates of physical problems and barriers in accessing health care may also be associated with an increased risk of premature mortality. In a Swedish study of over 27,000 individuals with autism, Hirvikovski et al. (2016) found that mean age of death was significantly earlier (~ 54 years) than in the general population (~ 70 years). The most frequent causes of death were nervous, circulatory, respiratory or digestive disorders and congenital malformations. Overall, death rates in males and females were similar, but women were more likely to die from endocrine disease, congenital malformations or suicide, and males from diseases of the nervous and circulatory systems. Premature death was most frequent in autistic individuals with intellectual disability (mean age of death 39 years), and in this group epilepsy was the most commonly reported cause of mortality.

### Gender

Lotter (1978), in his review of early follow-up studies noted that “among the children with the best outcome, there are very few girls”. Subsequent follow-up studies have also suggested that, compared with males, women seem to have poorer social and employment outcomes, higher rates of mood and anxiety related problems (Croen et al. 2015; Howlin et al. 2013; Moss et al. 2015; Taylor et al. 2015), and lower overall quality of life (Hong et al. 2016; Lever and Geurts 2016a, b). However, findings are compromised by the very small numbers of females involved in most of this research. Although more studies are now exploring gender differences in autism, much is still to be known about the presentation, needs and outcomes of autistic females (Halladay et al. 2015; Kirkovski et al. 2013; Mandy and Lai 2017). In particular, there are growing concerns about a possible gender bias in diagnosis, with suggestions that many women and girls fail to be correctly diagnosed because they show a somewhat atypical presentation of symptoms. For example, they are more likely to try to socialize than males, and special interests often have a more social content; women may also be better able to develop compensatory behaviors that mask their social difficulties (e.g. Dean et al. 2017; Duvekot et al. 2017; Lai et al. 2017; Mandy 2019). As standard autism diagnostic assessments make no allowance for possible gender differences, it has been suggested that females who met diagnostic criteria in earlier follow-up studies may have had more severe and observable difficulties and hence poorer outcomes. As yet, the potential impact of gender on outcome awaits findings from more systematic and large scale research. However, errors or delays in diagnosis of females, a lack of understanding of, or support for their difficulties, and the constant stress of trying to appear normal, may increase their risk of emotional and other problems (including eating disorders), while their social vulnerability exacerbates the risk of victimization and abuse (Fuentes et al. 2020; Happé and Frith 2020).

## The Impact of Environmental Factors

### Post School Support

Although individual characteristics clearly pay a crucial role in outcome, lack of adequate provision post-school, the absence of appropriately structured support or daily activities (Taylor and Selzer 2011), and the constant demands of ‘fitting-in” to a non-autism world (Hull et al. 2017) also have a major impact. In an early study of social outcomes in autism, Lord and Venter (1992) concluded that “the availability of support services is often one, if not the major factor in what happens to high-functioning young autistic adults”. They also noted that access to educational and employment facilities often depended more on *where* individuals lived than any other factors. Over the subsequent decades there has been a steady growth in specialized programs to assist young autistic adults gain access to higher education and to find and maintain employment. Many colleges and universities have developed support networks and/or modified teaching curricula in order better to meet the needs of autistic students (Elias and White 2018; Gillespie-Lynch et al. 2017), and there are some specific programs designed to assist transition to college (White et al. 2017). However, the general availability of such programs is scarce, and few have been systematically evaluated (Gelbar et al. 2014). Accounts by autistic students also indicate that the amount of support available varies widely; lack of understanding by and/or negative attitudes from teaching staff and/or other students are continuing problems, and social integration is often limited (Accardo et al. 2019; Anderson et al. 2018; Ashbaugh et al. 2017; Cage and Howes 2020; White et al. 2019). Many students with autism also remain uncertain whether to disclose their diagnosis to college authorities for fear of discrimination and hence do not take up supports that may be available (Gillespie-Lynch et al. 2017). Consequently, the numbers of autistic students successfully completing college or vocational courses remains low. Figures from the US National Longitudinal Transition study (Shattuck et al. 2012), involving 680 autistic youth, indicated that, two years after leaving school, only around 9% were enrolled in a vocational or technical programme, and among those in college only 12% were on full time courses; 35% had no participation in either education or employment.

Lack of vocational support post-school, or help to find appropriate employment, means that only a minority of autistic adults is able to find or retain work (Burgess and Chimera 2014). Even among cohorts of average or above average IQ, less than a third are in work and among those who are employed, jobs tend to be low-skilled, part-time and/ or poorly paid (Hedley et al. 2017). In a self-report survey of over 250 autistic adults, Gotham et al. (2015) found that, despite many having university degrees, around 60% were unemployed, a third did not live independently, and just under 40% relied on various benefits and supports. Failure to find employment has a major impact on many aspects of individuals’ lives, severely limiting opportunities for independent living, social integration and access to leisure and recreational activities. However, although supported employment schemes have been found to improve job finding and retention (see below) relatively few individuals have access to programmes of this kind.

### Family and Social Influences

There are few studies exploring the influences of family, parenting characteristics and/or parental mental health, or other wider familial or socioeconomic factors, on adult outcomes. There is some evidence that quality of mother–child relationship may be associated with later difficulties in adulthood (Woodman et al. 2015), and that adverse social experiences in childhood, such as bullying, are related to poorer self-reported quality of life (Hong et al. 2016). As most longitudinal studies to date involve families who are relatively well functioning (engagement in long-term research requires a considerable degree of organization and commitment) little is currently known about the possible negative impacts of familial or social adversity, or the potential long-term benefits of good social, financial and emotional support. Simonoff et al. (2019), in a longitudinal community study of autistic individuals (n = 126; mean age 23 years), found no impact on outcome of family variables, such as maternal mental health, parental educational level or neighborhood deprivation. In contrast, Farley et al. (2009), in a follow up study of 41 adults (mean age 32 years) with average or near average intellectual ability, reported more positive outcomes than for most other longitudinal cohorts, in terms of employment, social relationships and integration. Almost all the participants in that study had grown up in communities belonging to the Church of the Latter Day Saints in Utah, suggesting that the lifelong social support offered by such communities might play an important role in fostering social inclusion. However, in a subsequent population-based study in the same region (Farley et al. 2018; n = 162), involving individuals of lower cognitive ability as well as participants from the original cohort, rates of employment, independent living and social integration were no higher than in most other longitudinal studies.

## What is a “Good Outcome” in Autism?

In the conclusions to their latest follow-up, Farley et al. (2018) noted that, although the results were more negative than in their earlier study, the findings did not take into account the subjective experiences of participants, which may be more positive than objective data indicate. They highlight the need for researchers to listen to the voices of people with autism themselves (or to proxy reports for those who cannot self-advocate) and to pay far greater attention to the qualitative experiences of adults with autism. Thus, while most research is consistent in reporting poor outcomes for many autistic adults, it is important to recognize that definitions of a “good” social outcome or “good” quality of life, are based on “normative” criteria typically used in the general population (i.e. having a high level of social engagement, in work, living independently etc.). Rather belatedly, researchers have begun to recognize that concepts such as these may not always be relevant for people with autism (Ayres et al. 2018). Indeed, higher levels of employment and social engagement can come at the cost of poorer mental health and lower “subjective” quality of life (Bishop-Fitzpatrick et al. 2016; Moss et al. 2017). It is important, too, to recognize that not everyone with autism necessarily wishes to have close friendships or be in full time, competitive employment (McConachie et al. 2019). Thus, there has been increasing focus on the importance of the “person-environment fit” (Henninger and Taylor 2013; Lai et al. 2020) and the need to identify the characteristics of an “autism friendly” environment. These characteristics may involve similar, as well as different factors from those typically associated with quality of life. Potentially important factors include autism specific knowledge/training among caregivers; structured and individualized yet flexible support programs; purposeful occupation and/ or access to meaningful and enjoyable everyday life activities appropriate to capacity level; good physical and mental health; neighborhood support; supportive family contacts, and parental/ caregiver warmth (Billstedt et al. 2011; Bishop-Fizpatrick et al. 2018). Georgiades and Kasari (2018, pp. 716–717) have also called for a “Reframing” of notions related to “optimal outcomes”, highlighting the need for a much more comprehensive and inclusive definition that *captures diversity in individuals from across the autism spectrum, is based on within-individual growth over time, and describes progress on a wide range of variables and domains identified as meaningful by those living on the autism spectrum*. Autistic adults have also identified other factors that they consider important for an improved quality of life (McConachie et al. 2018, 2020). These include having a positive autistic identity; other people having a better understanding of autism; environmental modifications to help with sensory difficulties, and wider recognition of autistic people's contributions to society.

### Interventions for Adults with Autism

Many longitudinal studies have reported stability, or even improvements, in cognitive and other aspects of functioning over time. In the community cohort of Simonoff et al. (2019), for example, mean IQ increased from 78.6 in mid childhood to 84.8 in early adulthood. Such improvements led the authors to call for more research into factors, particularly educational and social experiences, that may promote cognitive and social development in early adulthood and beyond. They also highlight the need for better targeted interventions for individuals with autism across the life span. However, at the present time, there is a lack of empirically sound intervention studies for adults (c.f. National Institute for Health and Care Excellence (NICE) 2012) and there are no adult interventions that match the sophistication of intervention trials for young autistic children. In a systematic review of psychosocial interventions for adults, Bishop-Fitzpatrick et al. (2014) found that only 13 out of the 1217 studies reviewed were of adequate methodological rigor, and most focused on applied behavior analysis or social cognition training. Brugha et al. (2015) also conducted a systematic review of 30 intervention trials for adults (over 400 additional studies were rejected due to methodological limitations). Most included studies (67%) were pharmacological trials, with the main focus being on the reduction of behavioral problems or autistic symptoms, such as repetitive or ritualistic behaviors; none focused on improving quality of life, social participation or emotional well-being. Lack of progress in adult intervention research is confirmed in a more recent review by Shattuck et al. (2020) who identified only 52 papers (i.e. < 1% of over 23,000 peer-reviewed studies on autism) that had a focus on support systems, services or intervention programs for autistic adults.

### Interventions to Improve Social Skills

Among the more rigorously evaluated adult interventions, those designed to enhance social skills are probably the most numerous (c.f. Gates et al. 2017). Reported improvements include greater understanding and improved use of social skills, typically assessed by proxy report and/or analogue measures. The PEERS programme (Laugeson et al. 2015) is also reported to result in more frequent social engagement beyond the intervention context. However, almost all these interventions involve mainly adolescents/young adults of average to low average IQ; very few include older adults or those with more severe cognitive disabilities (Gates et al. 2017; Ke et al. 2018). Most studies also suffer from substantial methodological limitations. For example, in the review by Bishop-Fitzpatrick et al. (2014) of interventions targeting social functioning, most studies were single case/case series or non-randomized control trials. Similarly, Pallathra et al. (2019) identified only 11 RCTs in this area (only one of which was rated as “strong”) conducted from 1980 to 2017. Moreover, even in trials reporting positive effects, there is little evidence of generalization to naturalistic, community settings (Bishop-Fitzpatrick et al. 2014; Pallathra et al. 2019; Spain and Blaney 2015). Few social skills programs take account of the dynamic nature of social interactions and the crucial need to recognize and respond appropriately to the immediate demands of the social context. Vermeulen (2015) argues that, since “context blindness” is a specific characteristic of autism, attempts to “train” social skills in an artificial clinic setting will always be of limited value. Sasson and Morrison (2017) also highlight the fact that “successful” social interaction depends not only on the autistic person, but on others’ perceptions, judgments, reactions and responses as well. In order to minimize the daily interpersonal issues faced by many autistic adults there is clearly a need for much greater focus on practical interventions that can help to enhance social functioning and improve social relationships in dynamic, *real-life* situations (Bishop-Fitzpatrick et al. 2018; Garcia-Villamisar et al. 2017; Hong et al. 2016; Pallathra et al. 2019).

### Transition Services and Support for Employment

One of the most striking findings from adult outcome studies is the very low employment rate, even among cohorts of average/above average IQ. Over the last few decades many attempts have been made to improve this situation, with several studies highlighting the need to focus on the development of work skills well before individuals leave school or college (Burgess and Cimera 2014; Jonsson et al. 2019; Schall et al. 2020; Wehman et al. 2019). Although there is some evidence that individuals who receive transition services in early adolescence are more likely to be in employment and to be earning higher wages as adults (Burgess and Cimera 2014), the methodological rigor of most transitional research is poor. The very wide range of programs described in the literature also means that it is impossible to identify the specific components of intervention that are most effective (Westbrook et al. 2014). Moreover, relatively few specialist autism transition schemes exist and not all participants find them helpful. For example, respondents in a recent qualitative study (Snell-Rood et al. 2020) criticized poor communication, inadequate or inappropriate planning and goal setting, and lack of availability of community services once the transition programme was over.

Post-transition, the provision of specialist supported employment has been found to improve employment prospects for autistic adults. Many of these schemes are based on the original TEACCH programme of Schopler et al. (1997, 1983) and aim to provide individuals with help to find and maintain work. Importantly, too, they offer guidance for organizations, work managers and colleagues to understand the needs and difficulties, as well as the positive assets and skills of autistic employees. Supported employment programs have been demonstrated to be superior to alternative employment models, such as sheltered workshops, in terms of job level, pay, quality of life, and cost-effectiveness (Chen et al. 2015; Hedley et al. 2017; Mavranezouli et al. 2014; Nicholas et al. 2017; Wehman et al. 2019). Despite their apparent success, however, much is still to be learned about the individual and environmental factors that can contribute to improved and more sustainable educational and employment opportunities (Bury et al. 2020; Chen et al. 2015). Meanwhile, too, only a tiny minority of adults with autism has access to interventions of this kind.

### Interventions to Improve Mental Health

Another main focus of interventions for autistic adults has been on ways of improving mental health. Behavioral, cognitive, and mindfulness-based techniques have been shown to be moderately effective for problems such as anxiety, depressive symptoms and social anxiety, although less so for severe depression (e.g. Bishop-Fitzpatrick et al. 2014; Gaigg et al. 2020; Russell et al. 2020; Spain et al. 2017; Sizoo and Kuiper 2017). The quality of most existing research is also limited. Sample sizes are generally small, and the wide heterogeneity of participants and treatment methods means that it is not possible to identify which particular approaches work best for which individuals, or which are the essential elements of these multi-modal programs (Blainey et al. 2017). For example, Hesselmark et al. (2014) found little difference between cognitive behavior therapy (CBT) and a recreational activity program for autistic adults (mean age 32 years) attending an outpatient psychiatric clinic. Although there was no observed decrease in psychiatric symptoms, participants in each group reported improvements in quality of life, which the authors suggest may be due to the common elements of both types of intervention-i.e. structure and being in a group setting.

The rather limited, albeit generally positive, findings from these trials have led to a call for autism-specific adaptions to standard CBT techniques (e.g. NICE 2012). However, at present, there are no empirically derived guidelines on how best to adapt standard practice. Spain and Happé (2019) stress the importance of systematically assessing the views of people with autism themselves about what it is they hope to gain from cognitive based therapies, and what aspects of treatment they find more or less useful.

There is also a number of other approaches that may help to reduce mental health problems and improve quality of life for individuals with autism. For example, there is evidence that facilitating access to a wider range of leisure programs may help to improve both cognitive and social skills and general quality of life (Bishop-Fitzpatrick et al. 2017; Garcia-Villamisar et al. 2017; Stacey et al. 2019). Research by Bishop-Fitzpatrick et al. (2018) has also indicated the importance of social support, and interventions to improve daily life skills, for reducing stress and improving quality of life.

Finally, the personal accounts of many autistic adults have highlighted the very negative impact that sensory and environmental factors can have on their lives. Modifications to the physical environment (e.g. adaptations to ambient sound, lighting, space or temperature); to the social milieu (e.g. reducing social demands or stressors; avoiding ambiguity), or to daily routines (e.g. increasing predictability; reducing uncertainty; developing specialized interests, aptitudes and skills), may all help to reduce stress and anxiety and improve general functioning and well-being (Fuentes et al. 2020). When emotional and/or behavioral problems arise, the focus should be on exploring possible environmental triggers, and making modifications, as needed, to the physical, sensory and/or social environment (Matson et al. 2011).

### Improving Physical Health

As noted above, there is an increased risk of physical problems in autism, which can result in chronic health conditions and even premature mortality (Cashin et al. 2018). In individuals of higher cognitive or verbal ability, recognition by services of the inflated risk of suicide, and the provision of psychotherapeutic and environmental supports to reduce anxiety and depression are vital. Improvements in basic medical care by care staff are needed to reduce rates of death associated with intellectual disability or physical or neurological conditions. Health services staff, across all out-patient and in-patient facilities, require training to understand the specific needs of people with autism and the many barriers—personal, environmental and administrative—that are preventing them from utilizing either regular healthcare checks, or treatment when problems arise (Camm-Crosbie et al. 2019; Mason et al. 2019; Vogan 2017). A recent workshop hosted by Autistica, a leading UK autism charity, brought together autistic adults, relatives, clinicians, managers, clinical commissioners, international researchers, and funders with the aim of establishing a clinical and research agenda for improving physical health and well-being. The main priorities identified included: the need for systematic study of barriers to effective healthcare; the identification of what constitutes an “autism friendly” health service; an online tool kit to facilitate the primary health care of autistic adults; a personalized annual health check programme; investigation of the specific health difficulties in, and potential treatments for, older adults (especially related to cardiovascular and gut problems), and engagement with the autism community to improve knowledge about factors that may improve health and well-being (Warner et al. 2019). The report also identified the need for investigation into older autistic people's use and experiences of residential facilities.

## Listening to the Voices of People with Autism

Perhaps one of the greatest changes that have taken place over the last 40 years is the realization that, in order to improve services and support for autistic people and their families, and to ensure that research funding better reflects their priorities, it is crucial to listen to their own voices. Although several high profile autistic individuals with autism have been active in promoting both the assets and needs of people with autism over several decades (Grandin 2008; Lawson 2001; Robinson 2008), the introduction, in DSM-IV, of the new category of Asperger’s disorder presented opportunities for many more articulate young adults to find their “autism identity”; enabled them to build virtual communities across the world, and ensured that their voices were heard in educational and employment services, research organizations and many other areas of life. Despite the removal of the diagnostic label of Asperger’s disorder in DSM-5, the initial introduction of this term drew global attention to the many skills of autistic individuals and today it is increasingly unacceptable for research projects to be funded, or be granted ethical permission, without the active involvement of individuals with autism or their family members (Fletcher Watson et al. 2019). The development of a modified scale to assess QoL in autism, for example, is an illustration of how researchers can actively involve autistic adults in the design of more appropriate measures, and in making decisions about their own lives (McConachie et al. 2018, 2020). There has been some criticism that this shift in emphasis is often “tokenistic” rather than reflecting full collaboration with the autism community (Benevides et al. 2020; Milton 2019). Nevertheless, it is largely due to the direct involvement of “experts by experience” that we now understand more, for instance, about possible ways of minimizing barriers to mental and physical health care (e.g. Mason et al. 2019); how transitional and employment services might be improved (Snell-Rood et al. 2020), or about the potential importance and benefits of repetitive behaviors (Manor Binyamini 2020). Admittedly, there is still a great deal more to be done to take full account of the experiences of autistic people and their families, and the factors that that affect their lives, both positively and negatively. Adapting services to meet the expressed needs of adults, across the wider autism spectrum, also has a long way to go.

## Ageing in Autism

Although follow-up studies of adults with autism have increased in number over recent decades, the age of participants in these cohorts remains relatively low (mean age generally below 40 years; Mason et al. 2020), and there has been very little investigation of ageing in autism (Sonido et al. 2020). In the “typically” ageing population it is well established that variable such as poverty, social isolation, lack of employment and poor physical health are among the most significant predictors of poor mental health and reduced quality of life and, of course, these are factors that characterize the lives of many autistic adults. However, at the present time we still know very little about the impact of older age on social, emotional, cognitive or physical functioning and many questions remain to be answered. For example, what cognitive and other changes occur in a condition, such as autism, where cognitive and social difficulties are present from early childhood; do people with autism age more rapidly than other individuals; or might autism actually protect against “age-typical” decline in cognitive and other functions? Some years ago, Bowler (2006) raised the possibility that the life-long challenges faced by many individuals with autism could offer certain cognitive advantages in older age. Although research remains limited, there is some evidence that mental health, behavioral problems and autism symptoms may improve in mid-adulthood, while overall IQ and ratings of Quality of Life tend to remain relatively stable (Howlin et al. 2014; van Heijst and Guerts 2015). There have even been suggestions that “brain plasticity” in autism could act as a buffer against Alzheimer’s dementia (Oberman and Pascual-Leone 2014). While there is little hard evidence to support this view, certain areas of functioning, notably verbal fluency, seem to be *less* prone to decline in older autistic adults than in the general population (Lever and Geurts 2016a, b). Other cognitive skills (i.e. processing speed, attention, working memory, cognitive flexibility and planning) tend to show similar trajectories in autism as in the general population (Lever and Geurts 2016a, b), while visual memory may show greater decline. Roestorf and Bowler (2016) have also reported improvements in prospective memory and expressive vocabulary, and stability in executive functioning skills in older adults (> 50 years) compared with those under 50, whereas these abilities declined in a “typical” age-matched comparison group. Self- reports also indicated a reduction in mental and physical health problems over the same time period. However, these positive findings are not consistent across studies, and some other research has indicated a relative decline with age in overall IQ and cognitive flexibility in autistic adults compared with controls (Powell et al. 2017).

At the present time almost all studies on ageing in autism are small scale, mainly cross-sectional, and contain few participants aged over 60 (Roestorf et al. 2019). Understanding of the cognitive, social and physical and mental health trajectories of older adults with autism remains limited and current findings need to be replicated in larger, longitudinal studies. Nevertheless, it is clear that current planning and provision for specialized services for this group are inadequate and there is an urgent need to develop, and systematically evaluate, effective interventions and support services for older autistic adults, in both clinical and community settings. Finally, it is important to investigate the individual, social and environmental variables that can enhance social integration, independence and well-being well into the later years.

## What is Needed to Improve the Lives of Autistic Adults Over the Next 40 Years?

Kanner, in his follow-up study of individuals first seen in the 1940′s noted:

This 30-year follow-up has not indicated too much concrete progress from the time of the original report, beyond the refinement of diagnostic criteria. There has been a hodgepodge of theories, hypotheses and speculations and there have been many valiant, well-motivated attempts at alleviation awaiting eventual evaluation. It is expected…that a next 30- or 20- year follow-up …will be able to present a report …of more hopeful prognosis than the present chronicle has proved to be” (Kanner 1973, p 187).

Unfortunately, despite many advances in research during the 50 years following this statement, prognosis for the majority of autistic adults remains poor. It is true that few individuals, at least in countries with good health and social care, now spend their adult lives in long-term psychiatric institutions, as was the case for many of the patients described by Kanner. Earlier diagnosis, better support for families, and much improved educational provision for autistic children also mean that many more adults are now living in the community. Moreover, many adults, although continuing to be affected by their autism, do succeed in finding work, are able to live independently, and maintain close and meaningful relationships with others. However, such achievements do not come easily. Whilst some individuals have access to specialist educational and employment support, in many cases jobs are often found only with the support of families. Opportunities to live independently also depend heavily on local provisions—and often, too, on parental determination and persistence. And, even if individuals do manage to find work, many are in jobs that are inappropriate for their level of skills and qualifications. Relatively few develop or sustain long-term intimate relationships or are married /in stable partnerships. The risk of mental health difficulties is high, and life opportunities and experiences for many adults remain limited. In particular, a shortage of autism- specific adult provision means that many individuals, including those of high intellectual ability, continue to be financially, emotionally and physically dependent on their families for support and living accomodation (Lord et al. 2020).

So, what steps are needed to ensure a more positive outcome for adults with autism and reduce the pressures on them and their families?

1. There is a need for support networks and services that are available to far greater numbers of adults and continue well beyond the early adult years. While transitional and supported employment programs show some evidence of success, such schemes are limited in number, scope and duration. Access to flexible and individualized clinical and/or social support, that traverses the gap from child to adult services, must become more readily available*.* Being able to turn to a familiar professional at times of stress or difficulty, rather than having to wait, sometimes for many months, for a referral to a new and unfamiliar service, may help to minimize or even avoid potential problems. Rapid access to advice and support also reduces the risk of problems escalating to the extent that intensive and expensive crisis care is required. Parents and siblings, too, may require ongoing advice and support, while partners and/or children of autistic adults may need help to understand and manage the challenges that living with someone with autism can sometimes involve.
2. Options for adult living need to be expanded to meet the needs of far more individuals and across a much wider range of age and ability. These extend from residential care for those with the greatest need, to sheltered/supported housing for individuals requiring moderate levels of support, to good quality accommodation, with variable and flexible levels of support, for adults capable of semi/independent living. Similarly, dedicated education, employment and social support systems should be more widely available, more extensive, and better adapted to individual needs. These include better coordinated provision for autistic individuals with severe intellectual or mental health difficulties; specialist employment services (for both autistic employees and their employers); improved access to a range of educational and leisure facilities, and the provision of adequate social, financial and personal support to facilitate social inclusion and enhance well-being.
3. It needs to be acknowledged that the quality of research on interventions for adults is far inferior to that for autistic children, especially very young children. Thus, although there is some evidence of potentially effective interventions to improve adult social functioning and mental health, the variability and complexity of the treatments involved means that we still know very little about the specific components of services or therapies that are crucial for success. Evidence of methodologically sound and ecologically valid interventions and services is limited, and far greater funding and dedicated research is required to redress the current imbalance between child and adult research and access to effective interventions.
4. While the heterogeneity of autism is widely recognized, we still have very little empirical evidence on how interventions need to be adapted to meet individual abilities and needs, or modified according to family, social and cultural circumstances.
5. Much existing intervention research has concentrated on the difficulties associated with autism. However, recently there has been a refocusing of attention on autistic skills and assets. In his 30 year follow-up Kanner highlighted how special skills or interests had helped several of his adult patients to find their particular “niche” in life and how they had earned recognition amongst their peers and/or employers by their often unique skills and areas of knowledge. Since then it has been recognized that attributes such as attention to detail, perseverance, and/or special interests in areas such as mathematics, science, computing, art, music, history, the natural world etc. are common to many adults with autism. For example, Temple Grandin and Greta Thunberg are two world-renowned autistic women who have been setting an example of how autistic passions and interests can lead to exceptional achievements, not despite of, but because of their autism. The practical challenge for the future is how to develop strategies, from childhood onwards, that can enable autistic individuals to foster these skills and interests in ways that can facilitate social integration, lead to greater achievements in education and employment, and enhance the quality of their lives (c.f. Clark 2016; Bury et al. 2020).
6. Finally, as well as being one of the major changes in approaches to autism over the past four decades, attention to the voices of autistic people and their families is likely to be one of the main forces for change in the decades to come. The refrain of “nothing about us without us” (Milton 2013) is being increasingly heard and, hopefully, will lead to improved services and better collaborative and participatory research. This should result in research agendas, and research funding, becoming more focused on issues that will have a significant impact on the lives and wellbeing of people with autism, both across the life span, and across the world (Hoekstra et al. 2018).

## Summary

In this paper I have tried to summarize many of the changes that have taken place in our understanding of adulthood in autism in the four decades since the publication of DSM-111. Although, over this time, there has been a number of significant changes in the general conceptualization of autism, improvements in social outcomes for autistic adults have been slower to occur. Identifying the reasons why only some autistic individuals make significant improvements over time has major implications for our understanding of the factors influencing the course of development from childhood to adulthood. To date, the precise role played by variables such as gender, symptom severity, co-occuring conditions, or family and social influences remains unclear. Although cognitive and language abilities are closely linked to prognosis, having a high IQ and well developed language is not enough to guarantee a positive outcome. It may be that the ability to function adequately in adulthood depends at least as much on the acceptance and support offered by families, educational, employment, medical and wider social and community services as on individual characteristics. It is also important to recognize that “neurotypical” views of what constitutes a “good” outcome are not necessarily endorsed by everyone in the autism community. This highlights the need to listen more closely to the voices of autistic adults- and those living with or caring for them- in developing the research and service priorities that will make a real difference to the quality of their lives in the years to come.
